# Perinatal paracetamol exposure in mice does not affect the development of allergic airways disease in early life

**DOI:** 10.1136/thoraxjnl-2014-205280

**Published:** 2015-04-04

**Authors:** Debbie C P Lee, Simone A Walker, Adam J Byrne, Lisa G Gregory, James Buckley, Andrew Bush, Seif O Shaheen, Sejal Saglani, Clare M Lloyd

**Affiliations:** 1 Leukocyte Biology Section, National Heart and Lung Institute, Imperial College London, London, UK; 2 Immunology Programme, Centre for Life Sciences, National University of Singapore, Singapore, Singapore; 3 Department of Respiratory Paediatrics, Royal Brompton Hospital, and National Heart and Lung Institute, Imperial College London, London, UK; 4 Centre for Primary Care and Public Health, Blizard Institute, Barts and The London School of Medicine and Dentistry, London, UK

**Keywords:** Asthma, Allergic lung disease, Paediatric asthma

## Abstract

**Background:**

Current data concerning maternal paracetamol intake during pregnancy, or intake during infancy and risk of wheezing or asthma in childhood is inconclusive based on epidemiological studies. We have investigated whether there is a causal link between maternal paracetamol intake during pregnancy and lactation and the development of house dust mite (HDM) induced allergic airways disease (AAD) in offspring using a neonatal mouse model.

**Methods:**

Pregnant mice were administered paracetamol or saline by oral gavage from the day of mating throughout pregnancy and/or lactation. Subsequently, their pups were exposed to intranasal HDM or saline from day 3 of life for up to 6 weeks. Assessments of airway hyper-responsiveness, inflammation and remodelling were made at weaning (3 weeks) and 6 weeks of age.

**Results:**

Maternal paracetamol exposure either during pregnancy and/or lactation did not affect development of AAD in offspring at weaning or at 6 weeks. There were no effects of maternal paracetamol at any time point on airway remodelling or IgE levels.

**Conclusions:**

Maternal paracetamol did not enhance HDM induced AAD in offspring. Our mechanistic data do not support the hypothesis that prenatal paracetamol exposure increases the risk of childhood asthma.

**Key messages**

**What is the key question?**

- Does paracetamol exposure during pregnancy or lactation lead to the development of allergic airways disease in early life?

**What is the bottom line?**

- Maternal exposure to paracetamol, either during pregnancy, or lactation, or both, does not affect development of house dust mite induced allergic airways disease in neonatal mice.

**Why read on?**

- This is the first mechanistic study to investigate a causal link between prenatal and early life paracetamol exposure and the subsequent development of allergic airways disease.

## Introduction

An association between paracetamol use in pregnancy and increased risk of early childhood wheezing was first reported in a large population-based birth cohort study several years ago.^1^ Paracetamol exposure in late gestation was subsequently associated with an increased risk of doctor-diagnosed asthma and elevated total IgE at 7 years, but not allergen skin test positivity, lung function or bronchial responsiveness; early gestational exposure was associated with an increased risk of asthma but not raised IgE.^2^ ^3^ It has been argued that confounding by unmeasured behavioural factors linked to paracetamol use is an unlikely explanation for these findings.^4^ Some evidence has recently been reported for interactions between maternal antioxidant gene polymorphisms and prenatal paracetamol exposure on childhood asthma risk, thus strengthening the likelihood of causality.^3^ ^5^ Since the original report, a body of epidemiological evidence from other birth cohort studies has accumulated,^6^ with most,^5^ ^7^ ^8^ though not all,^9^ confirming a link between prenatal exposure and subsequent asthma or wheezing.

Infant paracetamol exposure has also been linked longitudinally to later wheezing and asthma,^10–12^ independently of the effect of prenatal exposure^3^ and also to atopy.^3^ ^13^ While the most likely explanation for the association with asthma is confounding by early respiratory infection,^12–14^ we cannot rule out the possibility that paracetamol exposure, perhaps in synergy with viral infection, might promote persistence of wheezing.

While definitive evidence of a causal link can only come from trials in humans, primary prevention trials in pregnancy and infancy pose considerable challenges; one immediate way forward is to carry out experimental studies in animal models.^6^ In an adult mouse model it was demonstrated that paracetamol, in the equivalent of human therapeutic doses could, via its reactive metabolite, activate the transient receptor potential ankyrin-1 channel, leading to neurogenic airway inflammation, thus offering an alternative potential mechanism by which paracetamol might influence asthma pathogenesis.^15^ Using an established and characterised neonatal model of house dust mite (HDM) induced allergic airways disease (AAD)^16^ we have investigated whether maternal exposure to paracetamol during pregnancy and lactation promotes the development of AAD in the offspring.

## Materials and methods

### Animals and paracetamol administration

**Figure 1:**
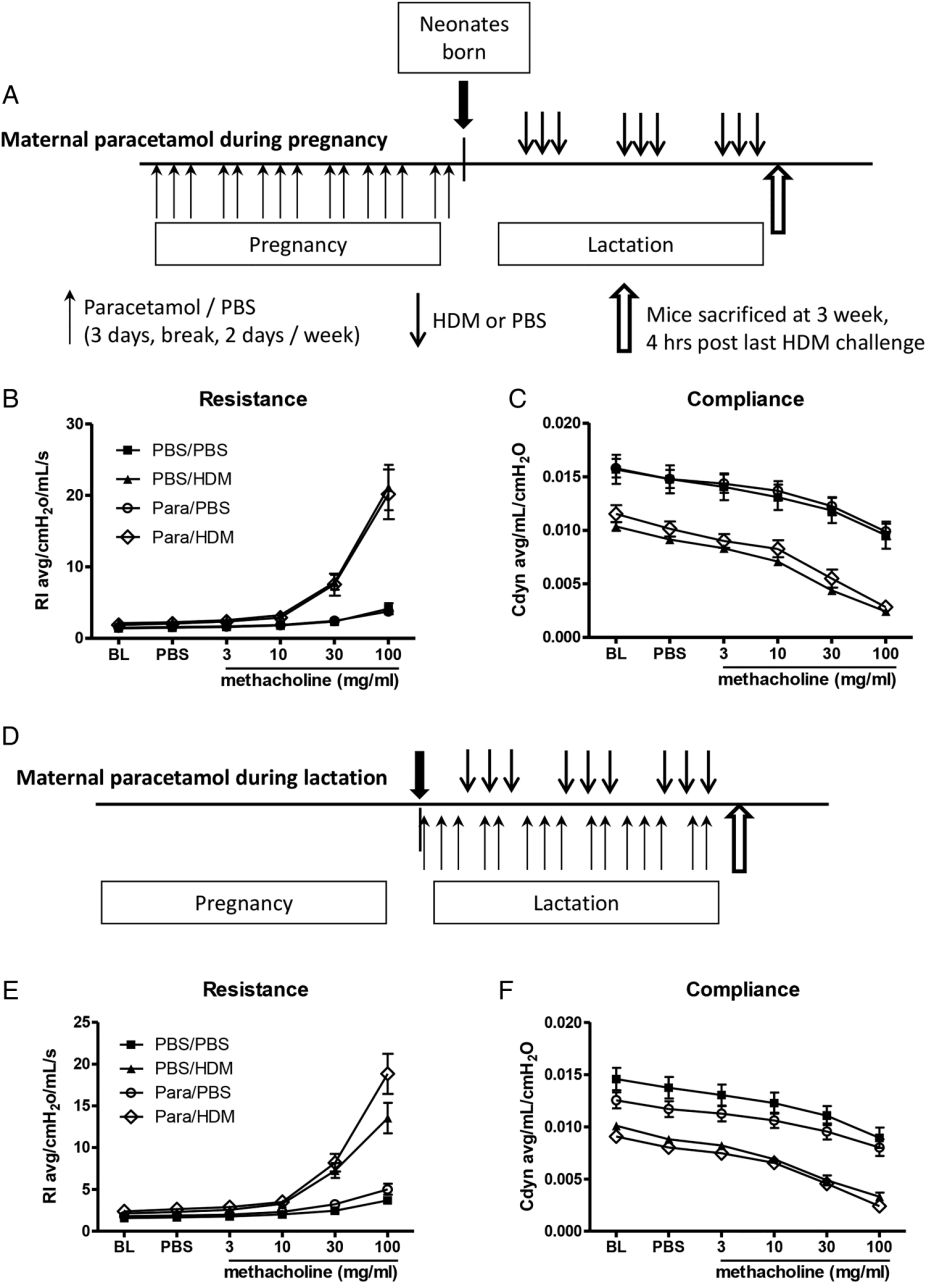
Similar airway hyper-responsiveness (AHR) following paracetamol exposure during either pregnancy or lactation. Experimental protocol for paracetamol exposure during pregnancy (A). Pregnant females were treated with 100 µL of 1.2 mg/mL liquid paracetamol by oral gavage 5 days a week, with a break on day 4 and day 7, during pregnancy (**↑**). Neonates were challenged with either house dust mite (HDM) or phosphate-buffered saline (PBS) intranasally on day 3 of life, three times a week for 3 weeks (**↓**). AHR measured as (B and E) lung resistance and (C and F) dynamic compliance to increasing doses of methacholine was determined 4 h after the last HDM challenge. Experimental protocol for paracetamol exposure during lactation (D). Female mice were mated and left for the duration of the pregnancy. On the day of birth, mothers were treated with 100 µL of 1.2 mg/mL liquid paracetamol by oral gavage 5 days a week, with a break on day 4 and day 7, during lactation, neonates were challenged with PBS or HDM from day 3 of life. Combined data of at least two experiments (n=10 for control mice and n=16–24 for HDM-exposed mice). No significant differences were found.

**Figure 2:**
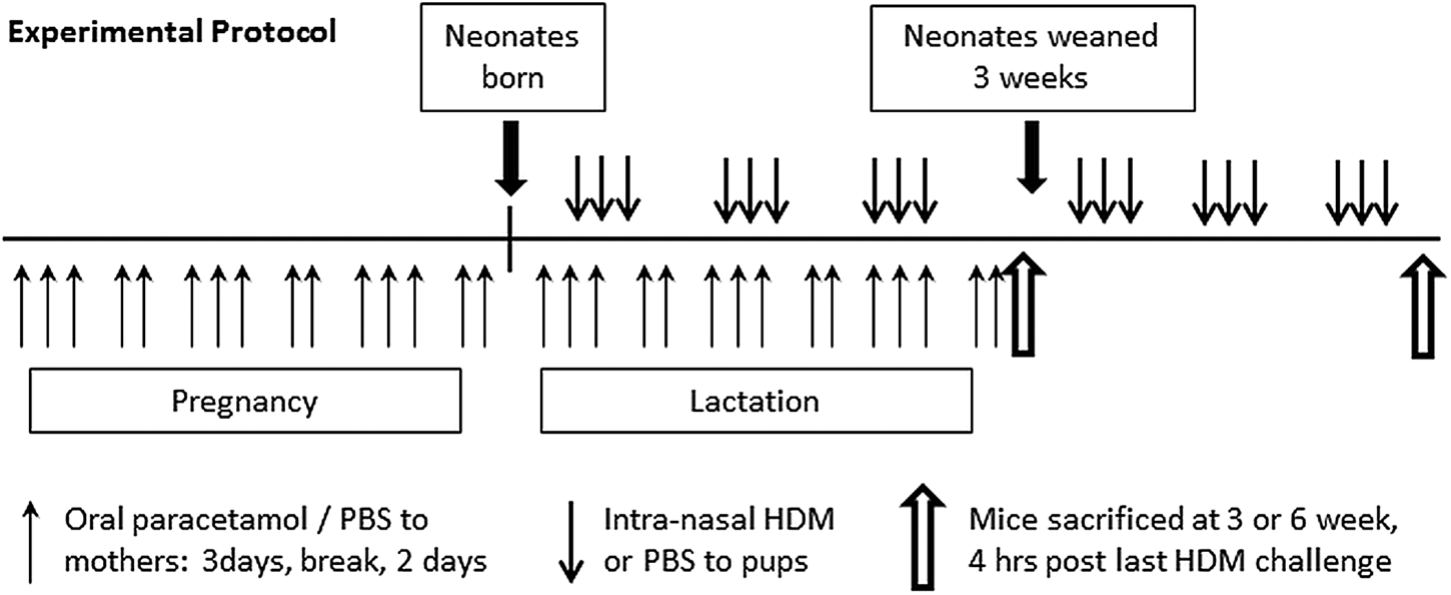
Experimental protocol for paracetamol exposure during pregnancy and lactation. Female BALB/c mice aged 6–8 weeks were treated with 100 µL of 1.2 mg/mL liquid paracetamol by oral gavage 5 days a week, with a break on day 4 and day 7, during pregnancy and lactation (**↑**). Neonatal mice were treated with either house dust mite (HDM) or phosphate-buffered saline (PBS) intranasally from day 3 of life, three times a week for either 3 weeks or 6 weeks (**↓**).

BALB/c mice were housed at Imperial College animal facility and used at 8–16 weeks of age. Female mice were administered 100 µL of liquid paracetamol (Calpol) (120 mg/5mLs paracetamol) or phosphate-buffered saline (PBS) by oral gavage either during pregnancy or lactation alone (figure 1), or both during pregnancy and lactation (figure 2). The dose of paracetamol was based upon the maximum recommended dose of 60 mg/kg/day for an average human. The potential effects of oral gavage in inducing stress during pregnancy were assessed by including a group of naïve pregnant females that underwent no treatment. Since there was no significant effect of oral gavage alone, data for PBS treated and naïve mothers were combined. In order to mimic findings from the epidemiological data which only showed effects with frequent and regular maternal paracetamol use, we administered paracetamol on 5 days every week. Litters were housed with their mothers until weaned at 3 weeks. All mice and litters were maintained in specific pathogen-free conditions and given food and water ad libitum. UK Home Office guidelines for animal welfare based on the Animals Scientific Procedures act 1986 were observed.

### Induction of AAD

Pups were exposed to 10 µg (10 µL of 1 mg/mL protein weight solution in PBS) of purified HDM extract (Greer Laboratories, Lenoir, North Carolina, USA) or PBS intranasally from day 3 of life for 3 days/week for 2 weeks, followed by 15 μg for up to 3 weeks or 6 weeks.^16^

### Measurement of AHR

Airway hyper-responsiveness (AHR) was measured as a terminal procedure 4 h after last allergen challenge in response to increasing doses of methacholine (3–100 mg/mL, Sigma, Poole, UK) in tracheostomised, anaesthetised mice using a Flexivent system (Scireq, Montreal, Canada) as described previously.^16^

### Cell recovery

#### Airway Lumen

Bronchoalveolar lavage (BAL) was performed using three aliquots of 0.3 mL PBS for 3-week-old mice and 0.4 mL PBS for 6-week-old mice, via a tracheal cannula. BAL fluid was centrifuged (200×g, 5 min at 4°C), supernatants were stored at −80°C for cytokine analysis and cells were resuspended in 0.5 mL complete media (Roswell Park Memorial Institute medium (RPMI)+10% fetal calf serum (FCS), 2 mM L-Glutamine, 100 U/mL Penicillin/Streptomycin).

#### Lung parenchyma

One lobe of lung tissue was mechanically chopped and incubated at 37°C for 1 h in complete media containing 0.15 mg/mL collagenase (Type D, Roche Diagnostics, Lewes, UK) and 25 μg/mL DNase (Type 1, Roche Diagnostics). Cells were recovered by filtration through a 70 μm nylon sieve, washed twice and resuspended in 1 mL complete media.

#### Serum

Blood was collected by cardiac puncture and transferred into a microtainer with serum separation gel (BD, Fischer scientific). Tubes were centrifuged at 14 000 RPM for 5 min to separate serum. Tubes were then stored at −20°C prior to analysis of immunoglobulins.

### Cytocentrifuge preparation and differential counts of Wright-Giemsa stained BAL and lung cells

Lung and BAL cells were applied to glass slides by centrifugation and stained with Wright-Giemsa (Thermo Fisher Scientific, Waltham, Massachusetts, USA). Percentages of macrophages, lymphocytes/mononuclear cells, eosinophils and neutrophils were determined under 40× magnification by counting cells in randomly selected fields and dividing this number by the total number of cells (400) counted. To obtain absolute numbers, this percentage was multiplied by the total number of cells recovered in lavage fluid and lung digest suspension. All cell counts were performed blind by the same observer.

### Analysis of cytokines

Cytokines were analysed in BAL samples and lung tissue homogenate supernatants. Lung tissue was homogenised at 50 mg/mL in Hank's balanced salt solution (HBSS) containing protease inhibitor tablets (Roche Diagnostics), centrifuged (800×g, 10 min) and the supernatant collected. BAL and lung homogenate cytokine levels were measured using paired antibodies for murine interleukin (IL) 4, IL-5, IL-25, IL-33 and interferon (IFN)-γ (BD Bioscience UK and R&D Systems, Abingdon, UK) in standardised sandwich ELISAs according to the manufacturer's protocol. Kits to measure IL-13 were purchased from R&D Systems. Paired antibodies for IgE (R&D systems) were used to measure serum IgE levels.

### Total lung collagen levels

Recently synthesised acid-soluble collagen was measured in the lung by biochemical assay (Sircol collagen assay; Biocolor, Belfast, UK) according to manufacturer's instructions and normalised for tissue weight.

### RNA extraction and qPCR

Total RNA was extracted from 50 mg to 100 mg of lung tissue by using a Qiagen RNeasy Mini Kit. Total RNA (1 μg) was reverse transcribed into cDNA using a High Capacity cDNA Reverse Transcription Kit (Life Technologies) as per the manufacturer's instructions. Real-time PCR reactions were performed using fast-qPCR mastermix (Life technologies) on a Viaa-7 (Life Technologies) instrument with TaqMan primer sets for murine amphiregulin, matrix metallopeptidase 2 (MMP-2), fibronectin, Found In Inflammatory Zone (FIZZ)1, vimentin, collagen-1α1 and Hypoxanthine-guanine phosphoribosyltransferase (HPRT) (Life Technologies) and gene expression was analysed using the change-in-threshold ΔΔCt- method.

### Statistical analysis

Data are expressed as median or mean±SEM. Group comparisons were performed using a non-parametric analysis of variance (ANOVA) test (Kruskal-Wallis), followed by a Dunn’s post test for multiple comparisons between groups. Graph generation and statistical analyses were performed using GraphPad Prism software (V.5.00; GraphPad).

## Results

### Maternal paracetamol exposure during pregnancy alone did not affect AHR in neonatal mice

In order to determine whether prenatal exposure to paracetamol during pregnancy altered lung function in neonatal mice exposed to HDM, pregnant females were treated with paracetamol on the 1st day of mating and throughout pregnancy. Paracetamol treatment was stopped on the day they gave birth. Offspring were exposed to intranasal PBS or HDM from day 3 of life, for 3 weeks (figure 1A). In utero paracetamol exposure alone did not result in worse lung function in HDM-exposed neonatal mice at weaning (3 weeks) (figure 1B, C).

### Maternal paracetamol exposure during lactation alone did not affect AHR in neonatal mice

Therefore, exposure during lactation was investigated to determine the influence of oral ingestion in the neonate via breast milk.^17^ Female mice were mated and left for the duration of the pregnancy. On the day of birth, mothers received the first dose of oral paracetamol which continued for 3 weeks. Pups were exposed to either intranasal PBS or HDM (figure 1D). HDM-exposed pups from paracetamol-treated mothers had similar AHR compared with those from PBS-treated mothers (figure 1E, F). There was no effect of paracetamol on inflammation in the BAL or lung (see online supplementary figure S1A,B), levels of IL-13 or IL-33 in the lungs (see online supplementary figure S1C,D), or total IgE and HDM-specific IgE (see online supplementary figure S1E, F).

### Maternal paracetamol exposure during pregnancy and lactation does not affect AAD in offspring

**Figure 3:**
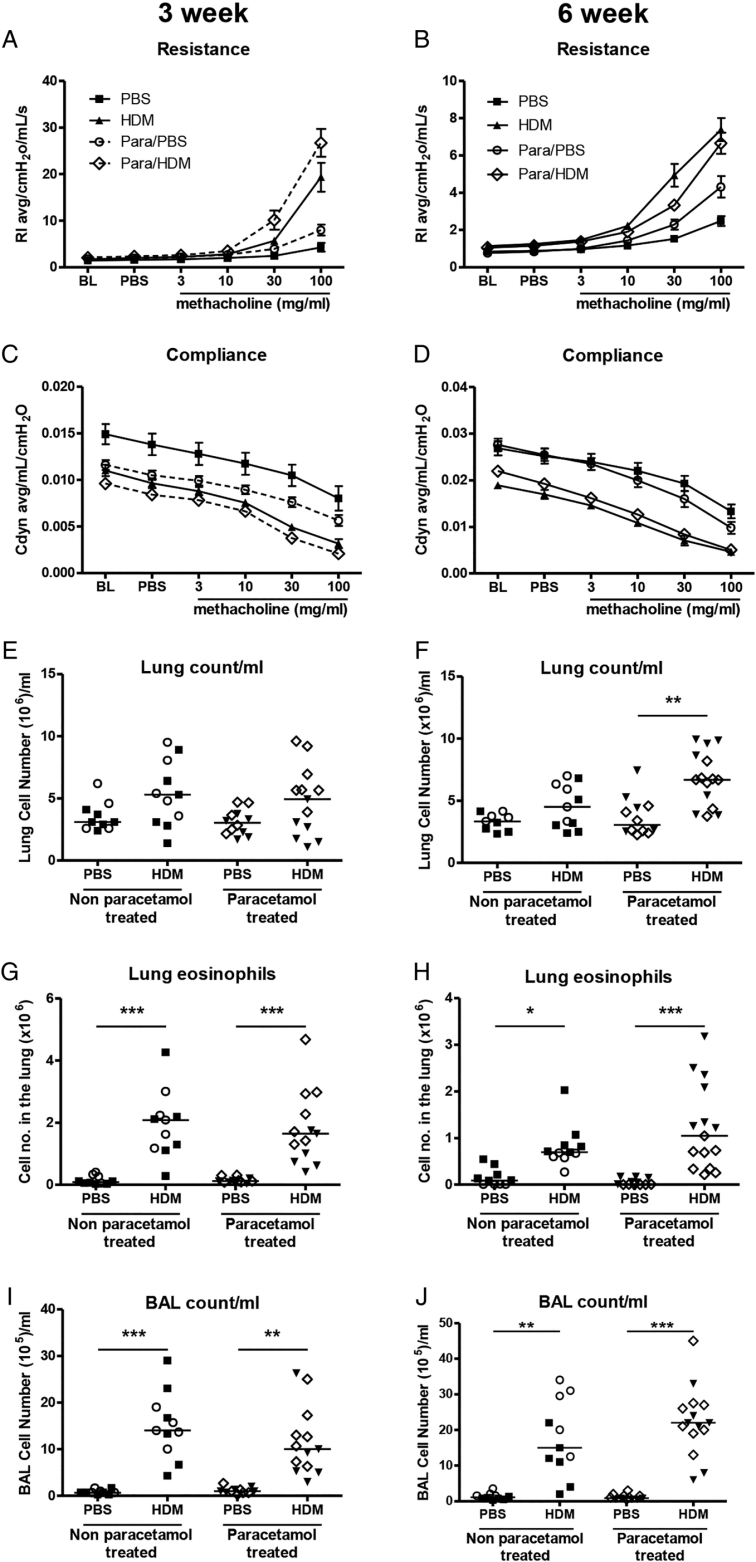
Allergic airways disease characterisation in house dust mite (HDM)-exposed neonatal mice from paracetamol-treated mothers. Airway hyper-responsiveness (AHR) in response to increasing doses of methacholine measured as lung resistance (A and B) and compliance (C and D) following 3 weeks or 6 weeks of HDM or phosphate-buffered saline (PBS) exposure, was determined in pups from either paracetamol-treated or non-paracetamol-treated mothers. Total inflammatory cells in the lung (E and F), total number of lung eosinophils (G and H) and total inflammatory cells in the bronchoalveolar lavage (BAL, I and J) were assessed at 3 weeks and 6 weeks. Combined data from two experiments (○ naïve females and ▪ PBS treated females) or paracetamol-treated mothers (◊ and ▾); (n=10–12 for control mice and n=11–15 for HDM-exposed mice). Horizontal bars represent median. There were no statistically significant differences between neonates exposed to paracetamol and those without paracetamol exposure at any time point.

As paracetamol exposure during pregnancy or lactation alone had no effect on neonatal AAD, additive effects of perinatal exposure during pregnancy and lactation were determined in early life (3 weeks) and in young adulthood (6 weeks) (figure 2). HDM-exposed pups from mothers treated with paracetamol during pregnancy and lactation had similar airway resistance and compliance at 3 weeks and 6 weeks compared with those from naïve or PBS-treated mothers (figure 3A–D). The total number of inflammatory cells recruited to the lung and BAL and eosinophils in the lung were unaffected by maternal paracetamol at 3 weeks and 6 weeks (figure 3E–J).

### Maternal paracetamol exposure during pregnancy and lactation does not alter neonatal inflammatory cytokines or IgE

**Figure 4:**
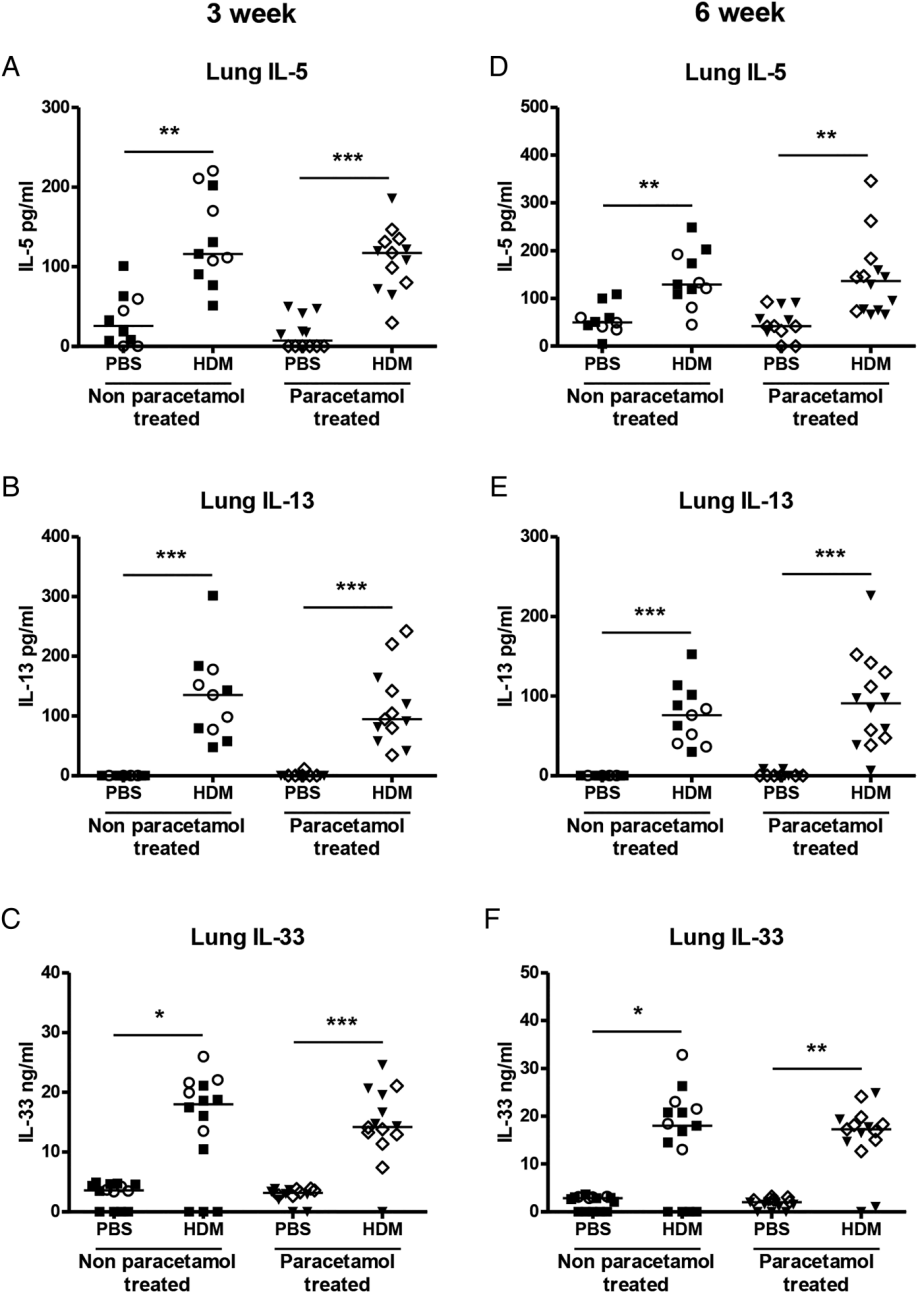
Levels of Th2 and innate cytokines in the lung. The levels of interleukin (IL) 5 (A and D), IL-13 (B and E) and IL-33 (C and F) in neonatal lung homogenate supernatant from non-paracetamol-treated (○ naïve females and ▪ phosphate-buffered saline (PBS) treated females) or paracetamol-treated mothers (◊ and ▾) were assessed by ELISA at 3 weeks and 6 weeks. Combined data from two experiments (n=10–12 for control mice and n=11–15 for house dust mite (HDM)-exposed mice). Significant differences between HDM-exposed and PBS-exposed neonates from non-paracetamol-treated or paracetamol-treated mothers are shown as **p<0.01 and ***p<0.001.

**Figure 5:**
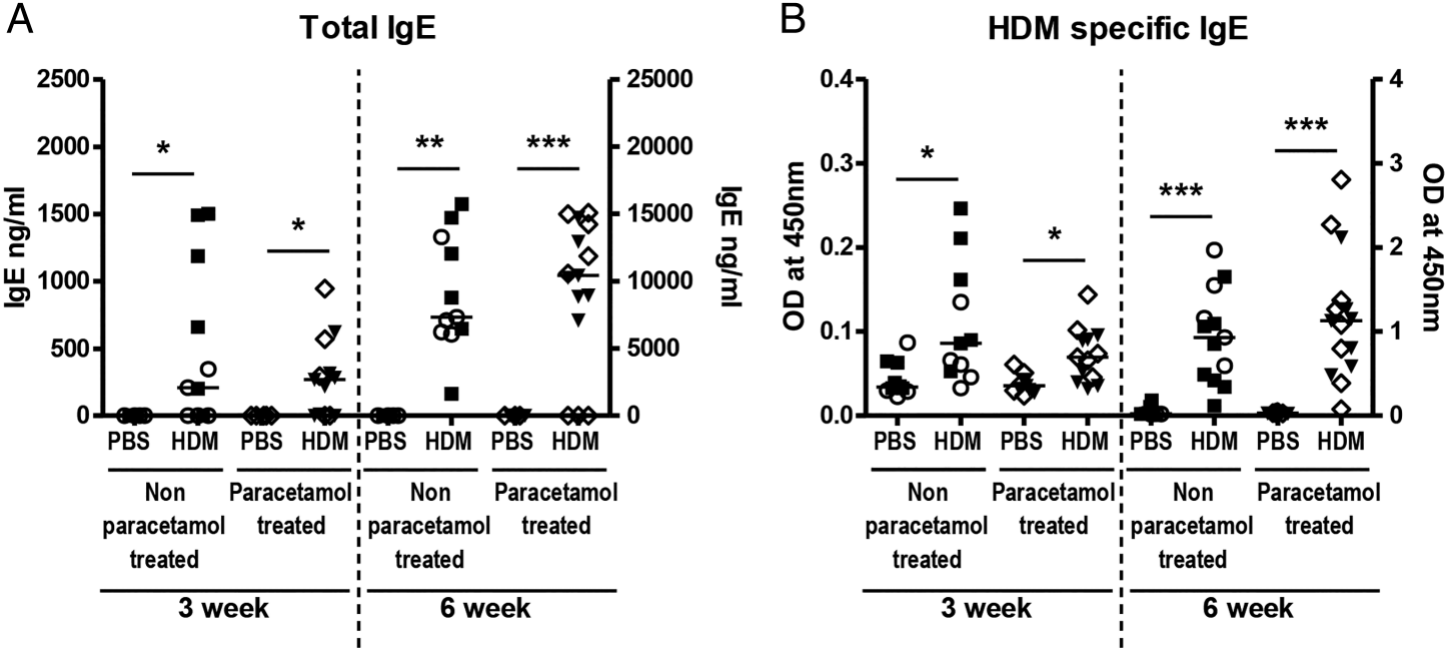
Total and house dust mite (HDM)-specific IgE levels. Serum from HDM-treated or phosphate-buffered saline (PBS)-treated neonatal mice from either non-paracetamol or paracetamol treated mothers was assessed by ELISA for total IgE (A) or HDM-specific IgE (B) following 3 weeks or 6 weeks of HDM or PBS exposure. Combined data from two experiments (○ naïve females and ▪ PBS-treated females) or paracetamol-treated mothers (◊ and ▾) (n=10–12 for control mice and n=11–15 for HDM-exposed mice). Significant differences between HDM-exposed and PBS-exposed neonates from non-paracetamol-treated or paracetamol-treated mothers are shown as **p<0.01 and ***p<0.001. ns, not significant.

The Th2 cytokines IL-5 and IL-13 were measured in BAL and lung and were significantly increased in HDM-exposed neonatal mice compared with PBS controls at 3 weeks, but maternal paracetamol did not alter levels of BAL (data not shown) or lung IL-5 or IL-13 (figure 4A, B). The same was true for the innate cytokine IL-33 (figure 4C). At 6 weeks levels of IL-5, IL-13 and IL-33 were still increased in HDM-treated groups, compared with controls (figure 4D–F), but there was no additional effect of maternal paracetamol. At 3 weeks and 6 weeks serum total IgE levels were significantly higher in HDM-exposed neonatal mice compared with controls, but there was no impact of maternal paracetamol exposure on total IgE levels (figure 5A). The same was also true for HDM specific IgE levels following 3 and 6 weeks of HDM exposure (figure 5B).

### Maternal paracetamol did not affect airway remodelling in HDM-exposed neonatal mice

**Figure 6:**
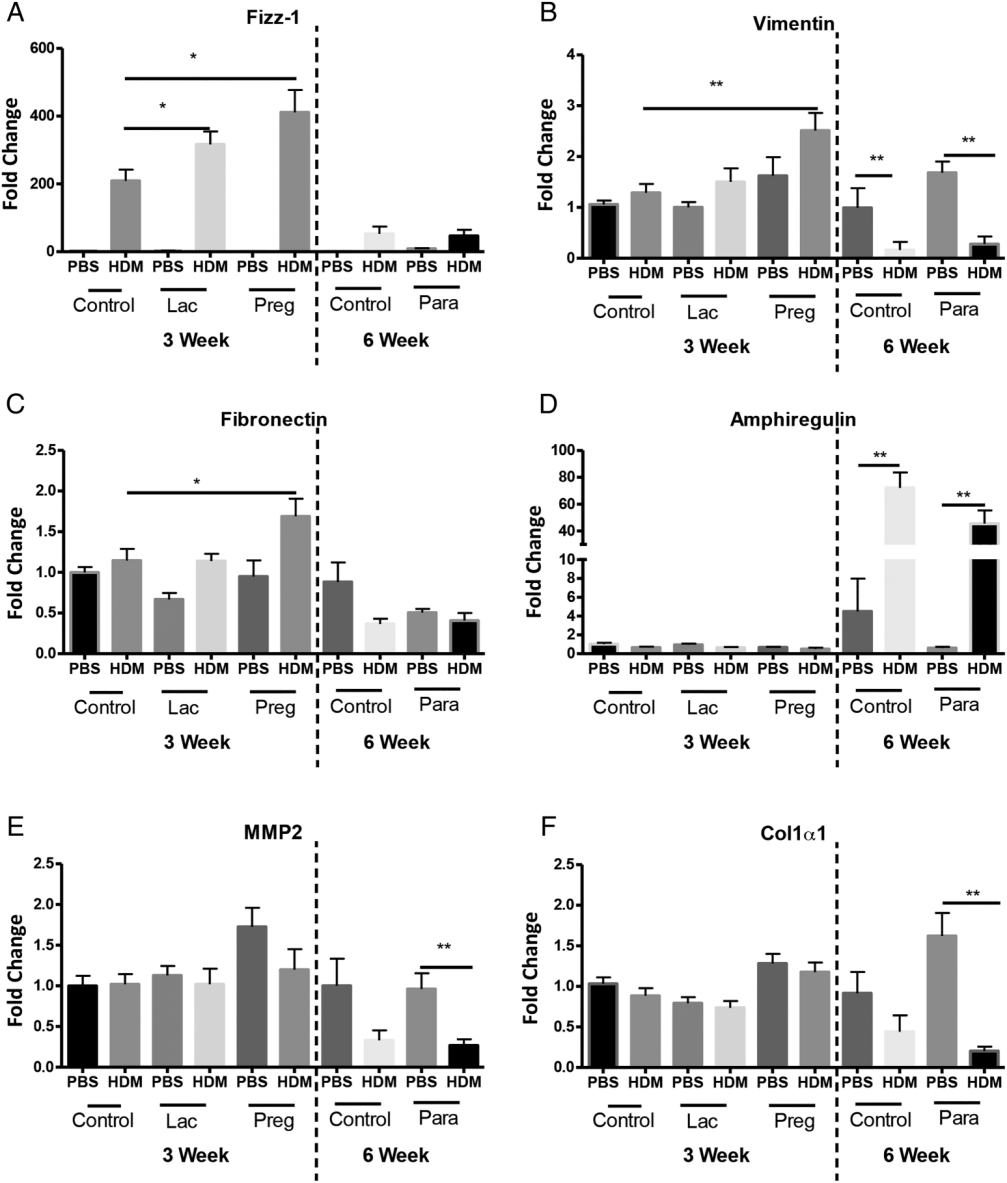
Increased levels of airway remodelling gene expression in house dust mite (HDM) neonatal mice from paracetamol-treated mothers. RNA was extracted from lungs from 3-week-old and 6-week-old neonatal mice exposed to HDM or phosphate-buffered saline (PBS), from mothers treated with paracetamol or PBS during pregnancy alone or lactation alone. mRNA levels for Found In Inflammatory Zone (FIZZ)1 (A), vimentin (B), fibronectin (C), amphiregulin (D), matrix metallopeptidase 2 (MMP)-2 (E) and collagen-1α1 (F) are shown as fold induction normalised to the control group, PBS-exposed neonates from PBS-treated mothers. Data represents three individual experiments (3 week data) or one representative experiment (6 week data). Significant differences between HDM exposed neonates from control or paracetamol-treated mothers and between PBS control mice are shown as *p<0.05 and **p<0.01.

**Figure 7:**
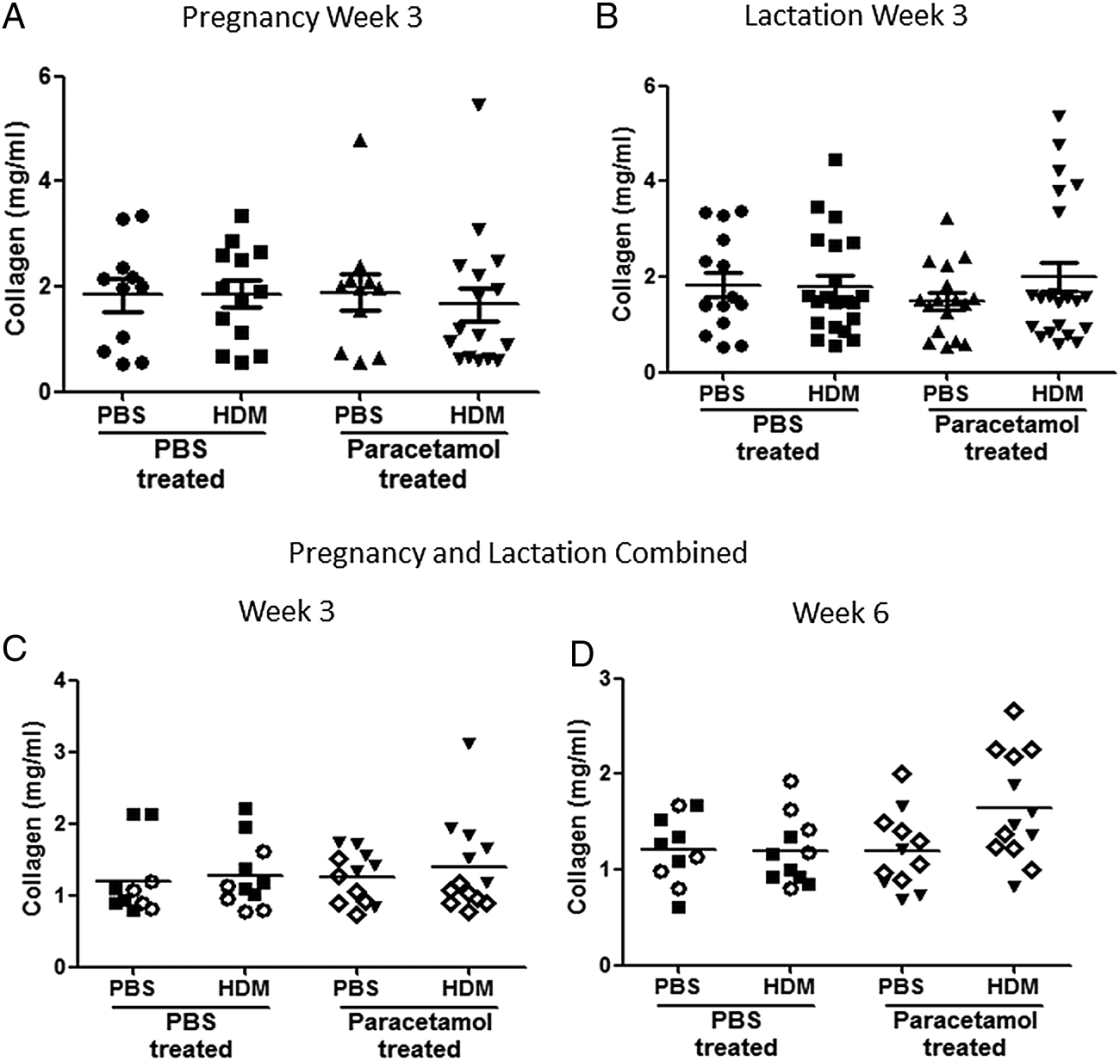
Similar levels of lung collagen in all house dust mite (HDM)-exposed neonatal mice, regardless of paracetamol exposure. Total lung collagen was measured in lung supernatants from neonatal mice treated with HDM or phosphate-buffered saline (PBS) for 3 weeks or 6 weeks, from mothers exposed to paracetamol during pregnancy or lactation only, and during both pregnancy and lactation using the Sircol biochemical assay. Combined data from at least two experiments (n=10 for control mice and n=16–24 for HDM-exposed mice). Horizontal bars represent median, *p<0.05 and **p<0.01.

Increased subepithelial reticular membrane thickness is an early feature of airway remodelling in preschool wheeze^18^ and childhood asthma,^19^ we therefore analysed mRNA levels of genes associated with remodelling including FIZZ1,^20^ fibronectin,^21^ vimentin,^22^ MMP-2,^23^ amphiregulin^24^ and collagen-1α1,^25^ from neonatal mice exposed to paracetamol during pregnancy alone (figure 1A), or lactation alone (figure 1D). Paracetamol treatment during pregnancy or lactation led to an upregulation of FIZZ1 expression in 3 week old HDM-treated neonates, in comparison to PBS-treated controls (figure 6A). Minimal changes were observed in the expression of vimentin and fibronectin, following 3 weeks of HDM in mice exposed to paracetamol in utero, in comparison to PBS exposed controls (figure 6B,C). However, these alterations did not lead to lung function changes (figure 1 and see online supplementary figure S2A–D). Importantly, the effect of paracetamol exposure was transient and was not observed following 6 weeks of HDM exposure. Expression levels of amphiregulin, MMP-2 and collagen-1α1 were unaffected by paracetamol exposure following 3 weeks or 6 weeks of HDM treatment (figure 6D–F). In order to determine whether differences in protein were present, total lung collagen was quantified by biochemical assay. Maternal paracetamol treatment during pregnancy, lactation, or both, had no impact on collagen levels in neonatal mice exposed to HDM for 3 weeks (figure 7A–C) or in young adulthood at week 6 (figure 7D).

## Discussion

To our knowledge, this is the first mechanistic study to investigate a causal link between maternal paracetamol intake and asthma in early life. We have provided direct in vivo evidence that maternal paracetamol exposure either during pregnancy, lactation, or during both, does not affect AHR in offspring exposed to inhaled HDM. There was also no enhanced inflammation, Th2 cytokines or increased levels of serum total or HDM-specific IgE. Although expression of early remodelling genes FIZZ1 and fibronectin were increased at weaning (3 weeks) following in utero paracetamol exposure, there was no associated change in protein (total lung collagen) or lung function, and the differences were not maintained into later life (6 weeks).

An epidemiological association between paracetamol exposure during pregnancy and wheezing in early childhood was first demonstrated over a decade ago in the Avon Longitudinal Study of Parents and Children (ALSPAC) birth cohort.^1^ Frequent paracetamol use, particularly in the latter half of pregnancy, was associated with persistent wheeze in children at age 3.5 years and asthma at age 7 years;^1^ ^2^ the relation with asthma was independent of the association between infant paracetamol exposure and asthma.^3^ Many other birth cohort studies have since shown a link between maternal paracetamol use during pregnancy and the development of wheezing or asthma in children.^6^

We first tried to mimic the effects of in utero exposure alone by administering paracetamol to female mice only during pregnancy, but in contrast to the epidemiological data showing a positive association between in utero paracetamol exposure and elevated serum IgE at 7 years,^2^ we saw no difference in either total or allergen-specific IgE levels. In addition, we found no effect of prenatal paracetamol exposure on any other features of AAD in our animal model. One explanation may be that the epidemiological association with maternal use in pregnancy is explained by residual or unmeasured confounding factors. For example, few cohorts had information on indication for use. A recent, large prospective study designed to look at the effects of maternal and infant paracetamol ingestion and subsequent asthma has only shown an association between prenatal paracetamol and asthma in early childhood, the effect was not sustained until mid-childhood.^26^ In order to see whether even a short-term increase in susceptibility to AAD was present following maternal paracetamol exposure, we assessed pups at 3 weeks and 6 weeks of age. In keeping with the findings from Sordillo *et al*, we saw a transient increase in expression of remodelling genes only at 3 weeks. However, there was no enhanced effect on lung function, inflammation or sensitisation apparent from maternal paracetamol at either 3 weeks or 6 weeks.

Another issue is whether infant ingestion may impact subsequent asthma development. One epidemiological study demonstrated that paracetamol ingestion by infants in the 1st year of life was associated with troublesome lower lung symptoms independently of lower respiratory tract infections in the first 3 years. There was no association between paracetamol ingestion during the 1st year and asthma at 7 years of age, or between maternal paracetamol ingestion during pregnancy and asthma or troublesome lower lung symptoms.^27^ Similarly, a much larger prospective study has shown adjustment for respiratory infections in early life substantially reduces the association between infant paracetamol intake and subsequent asthma.^26^ However, evidence from other epidemiological studies on whether the link between infant use and asthma is confounded by respiratory infection is conflicting.^6^ ^12^ Paracetamol can be rapidly secreted into breast milk and is detectable at higher concentrations in the milk compared with maternal plasma.^17^ Furthermore, paracetamol metabolites detected in the urine from babies from breastfeeding mothers, differ significantly from those detected in the urine of healthy adults who have ingested paracetamol. Neonates have significantly higher levels of paracetamol in their urine compared with adults, and adults have greater levels of urinary paracetamol sulfate,^17^ confirming that the pharmacokinetics and pharmacodynamics of paracetamol differ substantially in murine^28^ and human neonates and infants compared with children and adults.^29^ As it is technically very difficult to perform oral gavage in newborn mice, the importance of oral paracetamol ingestion in early life on subsequent AAD was indirectly determined in our study by administering paracetamol to mothers only during lactation and assessing AAD just prior to weaning at 3 weeks. However, we did not see any effect of oral ingestion via breast milk on the development of AAD in 3 week-old mice. There was also no long-term effect after weaning in mice exposed to HDM for 6 weeks. Paracetamol exposure did not alter inflammation or Th2 cytokines and levels of IgE were also unaffected.

Various genes have now been identified as important regulators of bronchial subepithelial basement membrane thickening and as such we analysed the mRNA expression levels of several fibrotic and early remodelling genes. Interestingly, we found there was a very small (less than twofold), but statistically significant increase in the mRNA levels of fibronectin and vimentin in allergen-exposed mice from mothers treated with paracetamol during pregnancy, but not during lactation; whereas FIZZ1 was upregulated after paracetamol exposure during lactation or pregnancy. FIZZ1 is induced in the lung following ovalbumin or *Alternaria* challenge,^20^ ^30^ while fibronectin has been shown to be essential for the development of ovalbumin-induced airway fibrosis and AHR.^20^ Vimentin and fibronectin are also associated with airway collagen deposition and airway remodelling.^21^ ^22^ Despite the transient increase in the expression of these genes we found no change in protein levels of lung collagen in HDM-treated neonatal mice exposed to maternal paracetamol.

We acknowledge that the findings from a murine model may not be directly applicable to the human situation. However, murine models have been used extensively to study paracetamol toxicity.^31^ Furthermore, the transient changes with paracetamol in FIZZ1, fibronectin and vimentin suggest that paracetamol crosses the placenta. There is no literature on whether paracetamol crosses into breast milk of lactating mice, but conversely, there is no evidence to suggest that breast milk metabolism in mice is significantly different from humans. To our knowledge, this is the first mechanistic investigation of a direct effect of paracetamol exposure either in utero, or orally via breast milk, or at both times, on neonatal HDM-induced AAD. The strengths include; (1) use of an age-appropriate model with inhaled allergen challenge, (2) administration of maternal paracetamol with a similar frequency and dosage, equivalent to those in epidemiological studies shown to have effects and (3) absence of any confounding from respiratory infections. With these best possible conditions, there was no effect of maternal paracetamol on the development of any parameters of AAD in offspring either in early life (3 weeks of age) or later at 6 weeks. These data may help to demonstrate why a recent systematic review of studies looking at the effect of paracetamol in pregnancy and early life and subsequent asthma has concluded (A) the association between exposure during pregnancy and asthma in childhood is highly variable between studies and is not robust, and (B) the association between paracetamol ingestion in infancy and subsequent asthma is confounded by respiratory infections.^32^

## Supplementary Material

Web figures
