# Supplementary material for: Perinatal paracetamol exposure in mice does not affect the development of allergic airways disease in early life
Source: Thorax. 2015 Apr 4;70(6):528–36. doi: 10.1136/thoraxjnl-2014-205280 (PMC4453715; doi:10.1136/thoraxjnl-2014-205280)

Supplementary Figure 1 Inflammatory parameters following maternal paracetamol exposure during lactation. Females were mated and left for the duration of the pregnancy. On the day of birth, mothers were treated with 100 μ l of 1.2 mg/ml liquid paracetamol by oral gavage 5 days a week, with a break on day 4 and 7, during lactation. Neonates were challenged with either HDM or PBS intranasally on day 3 of life, three times a week for 3 weeks. The total number of inflammatory cells in the BAL (A) and lung (B), levels of IL-13 and IL-33 in the lung supernatant and the total IgE and HDM specific IgE response were measured after 3 weeks of HDM exposure. Horizontal bars represent median. Data representative of at least 3 experiments (n = 5-10 for control mice and n = 6-21 for HDM exposed mice).

Sup. Fig. 1

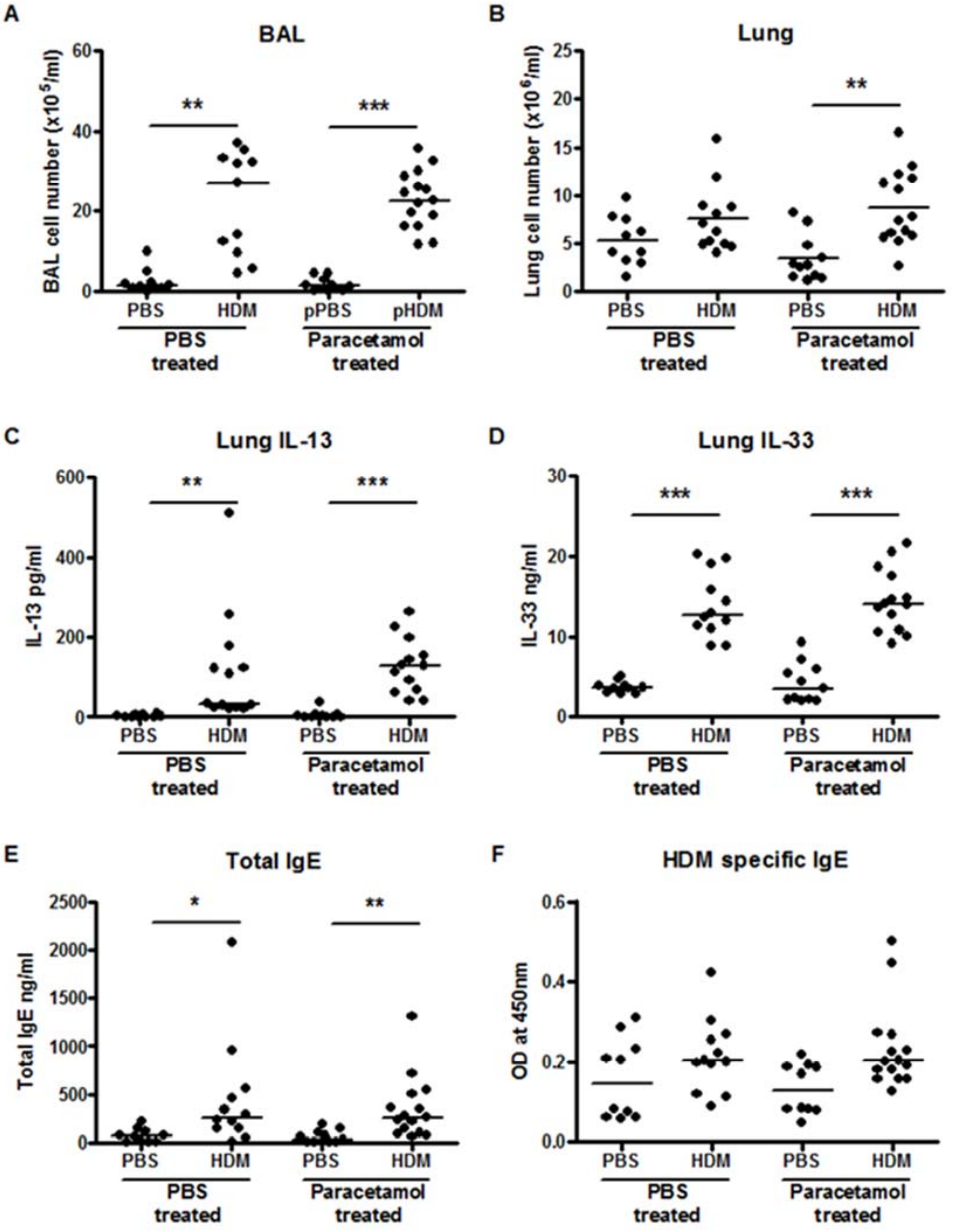

A
BAL
BAL cell number (x10⁵/ml)
60
40
20
0
**
***
PBS
HDM
pPBS
pHDM
PBS treated
Paracetamol treated
B
Lung
Lung cell number (x10⁶/ml)
25
20
15
10
5
0
**
PBS
HDM
PBS
HDM
PBS treated
Paracetamol treated
C
Lung IL-13
IL-13 pg/ml
600
400
200
0
**
***
PBS
HDM
PBS
HDM
PBS treated
Paracetamol treated
D
Lung IL-33
IL-33 ng/ml
30
20
10
0
***
***
PBS
HDM
PBS
HDM
PBS treated
Paracetamol treated
E
Total IgE
Total IgE ng/ml
2500
2000
1500
1000
500
0
*
**
PBS
HDM
PBS
HDM
PBS treated
Paracetamol treated
F
HDM specific IgE
OD at 450nm
0.6
0.4
0.2
0.0
PBS
HDM
PBS
HDM
PBS treated
Paracetamol treated

Supplementary Figure 2 Similar lung stiffness following maternal paracetamol exposure during pregnancy or lactation. The stiffness of the lung was analysed as tissue dampening (G) and tissue elastance (H) in neonates exposed to HDM for 3 weeks from mothers that were treated with paracetamol either during pregnancy (A and B) or during lactation (C and D). Combined data from two experiments (n = 10 for control mice and n = 16 for HDM exposed mice).

Sup. Fig. 2

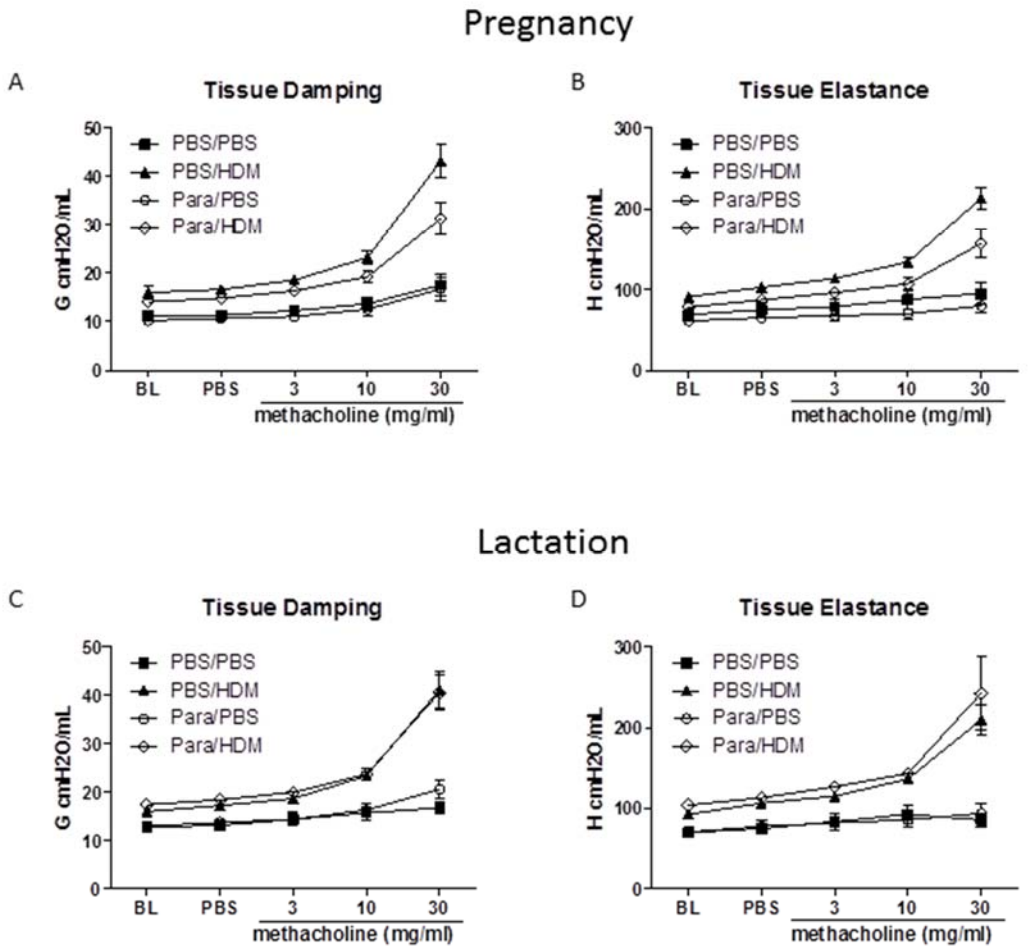

Supplement: Web figures [file thoraxjnl-2014-205280-s1.pdf]
